# Prolonged Time from Diagnosis to Breast-Conserving Surgery is Associated with Upstaging in Hormone Receptor-Positive Invasive Ductal Breast Carcinoma

**DOI:** 10.1245/s10434-021-09747-9

**Published:** 2021-03-21

**Authors:** Natalie Hills, Macall Leslie, Rachel Davis, Marielle Crowell, Hiroyasu Kameyama, Hallgeir Rui, Inna Chervoneva, William Dooley, Takemi Tanaka

**Affiliations:** 1 University of Oklahoma Health Sciences Center, College of Medicine, Oklahoma City, OK USA; 2 University of Oklahoma Health Sciences Center, College of Medicine, Stephenson Cancer Center, Oklahoma City, OK USA; 3 Department of Pathology, Medical College of Wisconsin, Milwaukee, WI USA; 4 Division of Biostatistics, Department of Pharmacology and Experimental Therapeutics, Thomas Jefferson University, Philadelphia, PA USA; 5 Department of Surgery, University of Oklahoma Health Sciences Center, College of Medicine, Oklahoma City, OK USA; 6 Department of Pathology, University of Oklahoma Health Sciences Center, College of Medicine, Oklahoma City, OK USA

## Abstract

**Background:**

Time to surgery (TTS) has been suggested to have an association with mortality in early-stage breast cancer.

**Objective:**

This study aims to determine the association between TTS and preoperative disease progression in tumor size or nodal status among women diagnosed with clinical T1N0M0 ductal breast cancer.

**Methods:**

Women diagnosed with clinical T1N0M0 ductal breast cancer who had breast-conserving surgery as their first definitive treatment between 2010 and 2016 (*n* = 90,405) were analyzed using the National Cancer Database. Separate multivariable logistic regression models for hormone receptor (HR)-positive and HR-negative patients, adjusted for clinical and demographic variables, were used to assess the relationship between TTS and upstaging of tumor size (T-upstaging) or nodal status (N-upstaging).

**Results:**

T-upstaging occurred in 6.76% of HR-positive patients and 11.00% of HR-negative patients, while N-upstaging occurred in 12.69% and 10.75% of HR-positive and HR-negative patients, respectively. Among HR-positive patients, odds of T-upstaging were higher for 61–90 days TTS (odds ratio [OR] 1.18, 95% confidence interval [CI] 1.05–1.34) and ≥91 days TTS (OR 1.47, 95% CI 1.17–1.84) compared with ≤30 days TTS, and odds of N- upstaging were higher for ≥91 days TTS (OR 1.35, 95% CI 1.13–1.62). No association between TTS and either T- or N-upstaging was found among HR-negative patients. Other clinical and demographic variables, including grade, tumor location, and race/ethnicity, were associated with both T- and N-upstaging.

**Conclusion:**

TTS ≥61 and ≥91 days was a significant predictor of T- and N-upstaging, respectively, in HR-positive patients; however, TTS was not associated with upstaging in HR-negative breast cancer. Delays in surgery may contribute to measurable disease progression in T1N0M0 ductal breast cancer.

Breast cancer is one of the most commonly diagnosed malignancies in the U.S.^1^,^2^ Staging at the time of diagnosis is an independent prognostic factor in breast cancer,^3^ and survival rates steeply decline with increasing stage.^4^ Clinical stage is determined by radiographic measurement of lesion size and disease spread to regional lymph nodes and distant organs.^5^ Subsequent pathologic staging is based on pathologic evaluation of tumor size and lymph node involvement in surgically resected lesions, along with the presence or absence of distant metastasis. Accordingly, upstaging is defined as a progressive discordance of tumor size, lymph node involvement, or distant metastasis between clinical and pathologic staging. Current literature suggests that dense breast tissue, palpable tumors, high grade, pleomorphic calcifications, lymphovascular invasion, and use of core-needle rather than vacuum-assisted biopsy are factors significantly associated with upstaging of ductal carcinoma in situ (DCIS) to invasive ductal carcinoma (IDC).^6^–^8^ A recent study suggested that prolonged time from diagnosis to surgery (time to surgery [TTS]) ≥30 days is also associated with increased risk of upstaging from DCIS to IDC (median 38 days, range 30–365 days).^8^ Additional studies have demonstrated delay in surgery in early-stage breast cancer is associated with increased mortality risk,^9^,^10^ raising the question of whether TTS is also associated with measurable disease progression; however, previous studies examining the association between TTS and disease progression of invasive disease have not found a link.^11^–^13^ This study aims to analyze the association between upstaging and TTS by hormone receptor (HR) status among women diagnosed with clinical T1N0M0 ductal breast cancer who received breast-conserving surgery (BCS) as their first treatment. We also analyzed other clinical and sociodemographic factors associated with upstaging.

## Methods

### Cohort

**Fig. 1:**
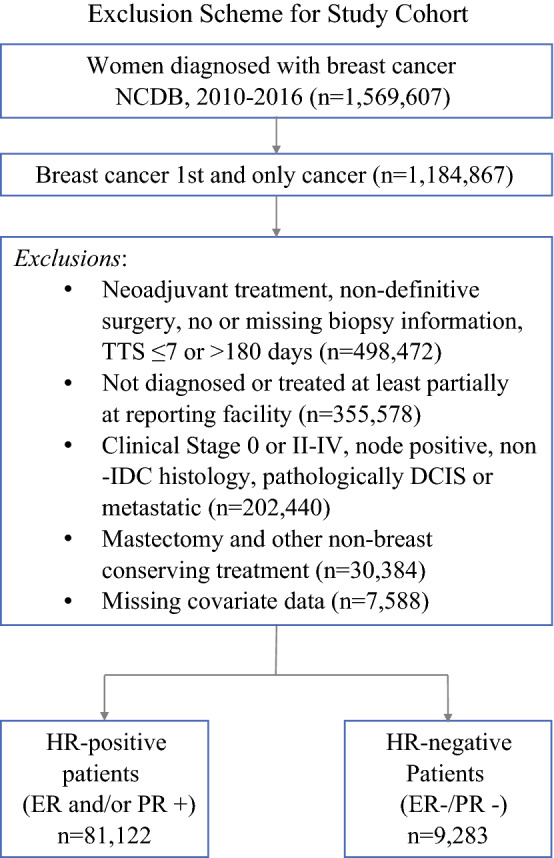
Exclusion scheme. *NCDB* National Cancer Database, *TTS* time to surgery, *IDC* invasive ductal carcinoma, *DCIS* ductal carcinoma in situ, *HR* hormone receptor, *ER* estrogen receptor, *PR* progesterone receptor

Women with clinical T1N0M0 ductal breast cancer diagnosed by needle or incisional biopsy between 2010 and 2016 who received BCS as their first definitive treatment were selected from the National Cancer Database (NCDB). The NCDB is a joint project of the Commission on Cancer (CoC) of the American College of Surgeons and the American Cancer Society, and all data used in this study were de-identified and met the criteria for exempt review by the University of Oklahoma Health Sciences Center Institutional Review Board (IRB# 7446). Women were selected based on a clinical stage of T1N0M0,^14^ ductal histology (International Classification of Diseases for Oncology, Third Revision [ICD-O-3] codes 8022, 8035, 8500–8503, 8523), and receipt of BCS as their first treatment (Surgery of the Primary Site codes 19–24). Only cases diagnosed and treated, all or in part, at the reporting facility were included in the final cohort. Cases with previous, concurrent, or subsequent malignancies, missing clinical or pathologic staging, no biopsy or missing biopsy information, neoadjuvant treatment, non-definitive surgery (i.e. excisional biopsy) as their first treatment, or with incomplete covariate data, were excluded (Fig. 1). Patients with pathologic stage IV disease were excluded from analyses due to the likelihood of undetected metastases present at the time of diagnosis rather than new clinically detectable metastases developing within the preoperative timeframe (*n* = 36, 0.03%). Additionally, the number of patients downstaged to pathologic in situ only tumors was insufficient (*n* = 47, 0.04%) to be examined as a separate outcome and was excluded from the analyses. Finally, patients who did not have any nodes examined or who had an unknown number examined were excluded due to discordance with the National Comprehensive Cancer Network/American Joint Committee on Cancer guidelines for determining pathologic stage.^14^,^15^

### Exposure

TTS was defined as the number of days between definitive diagnosis by biopsy and surgery and categorized by monthly intervals (i.e. ≤ 30, 31–60, 61–90, and ≥ 91 days). Patients who received surgery in 7 or fewer days were excluded from our study since receipt of surgery within 1 week of definitive diagnosis is unlikely in the modern setting (*n* = 1,824, 1.97%) and may be clinically reflective of greater medical urgency or unique circumstances. Additionally, patients with TTS over 6 months (180 days) were excluded from the study cohort due to exceptional delay (*n* = 127, 0.14%).

### Outcome

Upstaging was defined by two separate measures: T-upstaging as an increase in tumor size from clinical T1 to pathologic T2 or higher, and N-upstaging as a change in nodal status from clinical N0 to pathologic N1 or higher.

### Definitions

The cohort was grouped into two categories according to HR status (i.e. estrogen and/or progesterone)—HR-positive or HR-negative. HER2 status was determined using collaborative staging site-specific factor,^15^,^16^ which summarizes the results of immunohistochemistry and gene-amplification test (fluorescence in situ hybridization or chromogenic in situ hybridization) scoring of HER2 status as HER2-positive, HER2-borderline/equivocal, or HER2-negative. Patient race/ethnicity was categorized as White, Black, Hispanic, or other (Asian, Pacific Islander, American Indian/Eskimo/Aleutian), and age was dichotomized by the average age of menopausal onset (≤50 or >50 years), since menopausal status at the individual level was unavailable in the NCDB. Number of nodes examined during surgery was categorized as 1, 2–3, 4–5, or ≥6. Location of tumor was classified according to ICD-O-3 codes as nipple/central portion (50.0, 50.1), upper inner quadrant (50.2), lower inner quadrant (50.3), upper outer quadrant (50.4), lower outer quadrant (50.5), axillary tail (50.6), or overlapping/other (50.8, 50.9).

### Statistics

Patient characteristics were summarized by frequency and proportion for both T- and N-upstaging. For continuous variables, the median, range, first quartile (Q1), and third quartile (Q3) were used to summarize their distributions. Separate binary multivariable logistic regression models were used to examine the association between TTS and the outcome of either T- or N-upstaging, adjusted for dichotomized age, HER2 status, grade, tumor location, patient race/ethnicity, Charlson comorbidity score, and, for N-upstaging models, number of nodes examined. All statistical analyses were conducted using SAS software version 9.4 (SAS Institute, Inc., Cary, NC, USA), and graphs were generated using JMP version 14.

## Results

### Composition of the Cohort

Following exclusions, the final cohort included 90,405 patients diagnosed with clinical T1N0M0 ductal carcinoma who received BCS between 2010 and 2016, with 89.73% (*n* = 81,122) HR-positive and 10.26% (*n* = 9,283) HR-negative. The median age at diagnosis was 64 years (range 23–90; Q1: 56; Q3: 71) among HR-positive patients and 62 years (range 24–90; Q1: 55; Q3: 70) among HR-negative patients. The median TTS was 28 days (Q1: 20; Q3: 40) among HR-positive patients and 27 days (Q1: 19; Q3: 38) among HR-negative patients, and the proportion of TTS was also similar in both groups (≤ 30 days: 56.82%; 31–60 days: 36.88%; 61–90 days: 5.17%; ≥ 91 days: 1.13%, in HR-positive patients; ≤ 30 days: 60.09%; 31–60 days: 34.31%; 61–90 days: 4.61%; ≥ 91 days: 0.99%, in HR-negative patients). The proportion of T-upstaging grew with increasing TTS, from 6.6% of those with TTS ≤ 30 days to 9.59% of those with TTS ≥ 91 days among HR-positive patients; and from 10.77% of those with TTS ≤ 30 days to 13.04% of those with TTS ≥ 91 days among HR-negative patients (Table 1). Overall, 6.76% (*n* = 5,483) of HR-positive patients and 11.00% (*n* = 1,021) of HR-negative patients experienced upstaging from clinical T1 to pathologic T2 or higher (Table 1). The highest rates of T-upstaging in HR-positive patients were seen in tumors that were grade 3 or higher (13.99%), HER2-positive (9.80%), or located in the nipple/central portion of the breast (8.99%) and among Hispanic patients (9.06%). HR-negative patients who were ≤ 50 years of age (15.50%) or Hispanic (15.26%), or who had a Charlson Comorbidity Index ≥ 2 (13.32%) were more frequently T-upstaged (Table 1). With regard to N-upstaging, 12.69% (*n* = 10,298) of HR-positive cases and 10.75% (*n* = 998) of HR-negative cases were upstaged from clinical N0 to pathologic N1 or higher. The proportion of patients N-upstaged also grew with increasing intervals of TTS, i.e. from 12.69% of HR-positive patients with ≤ 30 days TTS to 16.34% of patients with ≥ 91 days TTS upstaged, and from 10.81% of HR-negative patients with ≤ 30 days TTS to 16.30% of patients with ≥ 91 days TTS. N-upstaging was most prevalent in HR-positive cases that were HER2-positive (14.83%), located in the axillary tail (18.06%) or nipple/central portion of the breast (18.93%), or had ≥6 nodes examined (35.38%), as well as among those who were Hispanic (16.59%), Black (15.15%), or ≤ 50 years of age (16.98%) [Table 1]. Higher proportions of N-upstaging occurred among HR-negative patients with tumors located in the nipple/central portion of the breast (14.78%), had ≥ 6 lymph nodes examined (35.45%), or who were Hispanic (14.79%) or ≤ 50 years of age (13.32%) [Table 1].

**Table 1:** Proportion of tumors upstaged by clinical or sociodemographic characteristics

	T-upstaging				N-upstaging			
	HR-positive		HR-negative		HR-positive		HR-negative	
	Not Upstaged	Upstaged	Not Upstaged	Upstaged	Not Upstaged	Upstaged	Not Upstaged	Upstaged
	*n* (%)	*n* (%)	*n* (%)	*n* (%)	*n* (%)	*n* (%)	*n* (%)	*n* (%)
Total Upstaged	75,639 (93.24)	5,483 (6.76)	8,262 (89.00)	1,021 (11.00)	70,824 (87.31)	10,298 (12.69)	8,285 (89.25)	998 (10.75)
TTS (days)								
≤ 30	43,055 (93.40)	3,043 (6.60)	4,977 (89.23)	601 (10.77)	40,249 (87.31)	5,849 (12.69)	4,975 (89.19)	603 (10.81)
31–60	27,885 (93.21)	2,030 (6.79)	2,822 (88.60)	363 (11.40)	26,158 (87.44)	3,757 (12.56)	2,858 (89.73)	327 (10.27)
61–90	3,869 (92.32)	322 (7.68)	383 (89.49)	45 (10.51)	3,649 (87.07)	542 (12.93)	375 (87.62)	53 (12.38)
≥ 91	830 (90.41)	88 (9.59)	80 (86.96)	12 (13.04)	768 (83.66)	150 (16.34)	77 (83.70)	15 (16.30)
Age group								
≤ 50	10,205 (91.29)	974 (8.71)	1,161 (84.50)	213 (15.50)	9,281 (83.02)	1,898 (16.98)	1,191 (86.68)	183 (13.32)
> 50	65,434 (93.55)	4,509 (6.45)	7,101 (89.78)	808 (10.22)	61,543 (87.99)	8,400 (12.01)	7,094 (89.70)	815 (10.30)
HER2								
Negative	68,763 (93.53)	4,754 (6.47)	6,655 (88.98)	824 (11.02)	64,324 (87.5)	9,193 (12.50)	6,734 (90.04)	745 (9.96)
Borderline	1,364 (91.30)	130 (8.70)	134 (88.16)	18 (11.84)	1,295 (86.68)	199 (13.32)	128 (84.21)	24 (15.79)
Positive	5,512 (90.20)	599 (9.80)	1,473 (89.16)	179 (10.84)	5,205 (85.17)	906 (14.83)	1,423 (86.14)	229 (13.86)
Grade								
1	31,000 (96.65)	1,075 (3.35)	188 (92.61)	15 (7.39)	29,105 (90.74)	2,970 (9.26)	187 (92.12)	16 (7.88)
2	34,854 (92.52)	2,816 (7.48)	1,973 (94.00)	126 (6.00)	32,255 (85.63)	5,415 (14.37)	1,881 (89.61)	218 (10.39)
≥ 3	9,785 (86.01)	1,592 (13.99)	6,101 (87.39)	880 (12.61)	9,464 (83.19)	1,913 (16.81)	6,217 (89.06)	764 (10.94)
Tumor location								
Upper inner quadrant	12,196 (93.38)	864 (6.62)	1,337 (88.66)	171 (11.34)	12,038 (92.17)	1,022 (7.83)	1,408 (93.37)	100 (6.63)
Lower inner quadrant	4,907 (94.29)	297 (5.71)	609 (89.17)	74 (10.83)	4,634 (89.05)	570 (10.95)	623 (91.22)	60 (8.78)
Upper outer quadrant	29,110 (93.17)	2,135 (6.83)	3,271 (87.76)	456 (12.24)	26,838 (85.9)	4,407 (14.10)	3,234 (86.77)	493 (13.23)
Lower outer quadrant	5,702 (92.73)	447 (7.27)	606 (91.27)	58 (8.73)	5,252 (85.41)	897 (14.59)	605 (91.11)	59 (8.89)
Axillary tail	292 (94.19)	18 (5.81)	NR	NR	254 (81.94)	56 (18.06)	NR	NR
Nipple/central portion	2,461 (91.01)	243 (8.99)	215 (93.48)	15 (6.52)	2,192 (81.07)	512 (18.93)	196 (85.22)	34 (14.78)
Overlapping/NOS	20,971 (93.41)	1,479 (6.59)	2,184 (89.91)	245 (10.09)	19,616 (87.38)	2,834 (12.62)	2,186 (90.00)	243 (10.00)
Race/ethnicity								
White	64,000 (93.59)	4,385 (6.41)	6,025 (89.63)	697 (10.37)	59,973 (87.70)	8,412 (12.30)	6,028 (89.68)	694 (10.32)
Hispanic	3,080 (90.94)	307 (9.06)	361 (84.74)	65 (15.26)	2,825 (83.41)	562 (16.59)	363 (85.21)	63 (14.79)
Black	6,188 (91.55)	571 (8.45)	1,670 (88.27)	222 (11.73)	5,735 (84.85)	1,024 (15.15)	1,675 (88.53)	217 (11.47)
Other	2,371 (91.51)	220 (8.49)	206 (84.77)	37 (15.23)	2,291 (88.42)	300 (11.58)	219 (90.12)	24 (9.88)
Charlson Comorbidity Index								
0	62,373 (93.34)	4,450 (6.66)	6,595 (89.11)	806 (10.89)	58,410 (87.41)	8,413 (12.59)	6,610 (89.31)	791 (10.69)
1	10,584 (93.05)	790 (6.95)	1,309 (89.11)	160 (10.89)	9,876 (86.83)	1,498 (13.17)	1,307 (88.97)	162 (11.03)
≥ 2	2,682 (91.69)	243 (8.31)	358 (86.68)	55 (13.32)	2,538 (86.77)	387 (13.23)	368 (89.10)	45 (10.90)
Number of nodes examined								
1					21,258 (92.25)	1,786 (7.75)	2,258 (94.75)	125 (5.25)
2–3					33,296 (89.50)	3,906 (10.50)	3,944 (92.65)	313 (7.35)
4–5					10,859 (86.85)	1,644 (13.15)	1,373 (88.98)	170 (11.02)
≥ 6					5,411 (64.62)	2,962 (35.38)	710 (64.55)	390 (35.45)

*HR* hormone receptor, *TTS* time to surgery, *NOS* not otherwise specified, *NR* not reportable

### Time to Surgery is Associated with Both T- and N- Upstaging in Hormone Receptor-Positive Patients

**Fig. 2:**
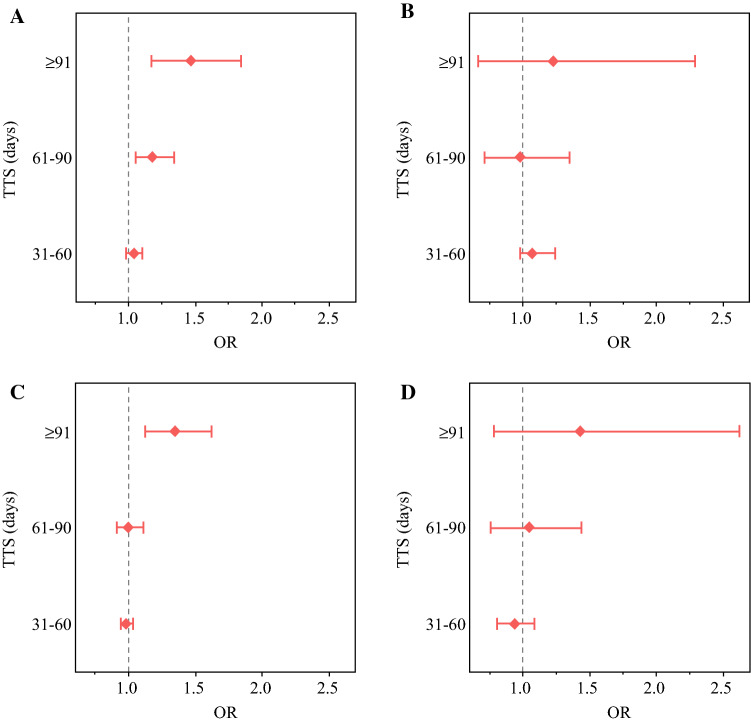
Association between TTS and T-upstaging (**a, b**) and N-upstaging (**c, d**) in HR-positive (left panel) and HR-negative (right panel). OR and CI were calculated relative to the reference (≤30 days TTS). *TTS* time to surgery, *HR* hormone receptor, *OR* odds ratio, *CI* confidence interval

Adjusting for the clinical and sociodemographic characteristics detailed in Table 1, HR-positive patients experienced a statistically significant increase in T-upstaging among those with 61–90 days TTS (OR 1.18, 95% CI 1.05–1.34) and ≥ 91 days TTS (OR 1.47, 95% CI 1.17–1.84) compared with those with *≤* 30 days TTS, while those with 31–60 days TTS did not experience a significant increase in risk (OR 1.04, 95% CI 0.98–1.1) [Fig. 2a]. However, TTS of any interval >30 days was not associated with T-upstaging in HR-negative patients after multivariable adjustment (Fig. 2b). Similarly, TTS was significantly associated with N-upstaging at ≥ 91 days TTS (OR 1.35, 95% CI 1.12–1.62) compared with TTS ≤ 30 days among HR-positive patients (Fig. 2c), but not at 31–60 or 61–90 days TTS. There was not a significant association between TTS and N-upstaging in the HR-negative model (Fig. 2d).

Other clinical and demographic factors were also associated with T- and N-upstaging, with statistical significance and effect sizes different according to HR status. Higher histologic grade was significantly associated with T-upstaging (OR 2.31, 95% CI 2.15–2.48 in grade 2; OR 4.58, 95% CI 4.21–4.98 in grade 3 or higher) and N-upstaging (OR 1.58, 95% CI 1.50–1.66 in grade 2; OR 1.80, 95% CI 1.68–1.92 in grade 3 or higher) in HR-positive patients, while among HR-negative patients, only grade 3 or higher was significantly associated with T-upstaging (OR 1.72, 95% CI 1.01–2.93) [Table 2]. HER2 status was not significantly associated with T-upstaging, but was associated with higher odds of N-upstaging among HR-negative patients (OR 1.63, 95% CI 1.01–2.62 in HER2-borderline; OR 0.51, 95% CI 1.27–1.78 in HER2-positive) [Table 2]. HR-positive tumors located in the nipple/central portion of the breast had higher odds of T-upstaging (OR 1.28, 95% CI 1.11–1.47) and N-upstaging (OR 1.41, 95% CI 1.27–1.57) than those located in the upper outer quadrant [Table 2]. The likelihood of N-upstaging grew substantially with increasing number of nodes examined. Patients who had > 6 removed/examined were more likely to be N-upstaged compared with those with only 1 node examined regardless of HR status (OR 6.17, 95% CI 5.77–6.59 in HR-positive patients; OR 9.6, 95% CI 7.71–11.97 in HR-negative patients). In terms of demographic variables, patients ≤ 50 years of age had statistically significant higher odds of T-upstaging (OR 1.18, 95% CI 1.09–1.28 in HR-positive patients; OR 1.42, 95% CI 1.18–1.72 in HR-negative patients) and N-upstaging (OR 1.26, 95% CI 1.18–1.34 in HR-positive patients; OR 1.19, 95% CI 0.98–1.46 in HR-negative patients), regardless of HR status. Additionally, HR-positive patients with a Charlson comorbidity index score of ≥ 2 were significantly more likely to be T-upstaged (OR 1.28, 95% CI 1.11–1.46) or N-upstaged (OR 1.13, 95% CI 1.01–1.27) than those without comorbidities (Table 2). Finally, regardless of HR status, Hispanic patients experienced significantly higher odds of T-upstaging (OR 1.28, 95% CI 1.13–1.45 in HR-positive patients; OR 1.40, 95% CI 1.06–1.85 in HR-negative patients) and N-upstaging (OR 1.27, 95% CI 1.15–1.40 in HR-positive patients; OR 1.38, 95% CI 1.02–1.86 in HR-negative patients). Similarly, Black HR-positive patients had higher odds of T-upstaging (OR 1.10, 95% CI 1.00–1.12) and N-upstaging (OR 1.10, 95% CI 1.02–1.18) than White patients (Table 2).

**Table 2:** Odds ratios for upstaging by clinical or sociodemographic characteristics

		T-upstaging				N-upstaging			
		HR-positive		HR-positive		HR-negative		HR-negative	
	Ref.	OR (95% CI)	*p* value	OR (95% CI)	*p* value	OR (95% CI)	*p* value	OR (95% CI)	*p* value
*HER2*			0.3421		0.7413		0.4148		< .0001
Borderline	Negative	1.15 (0.95–1.38)		1.21 (0.73–2.00)		1.01 (0.86–1.18)		1.63 (1.01–2.62)	
Positive		1.01 (0.92–1.11)		1.03 (0.86–1.22)		0.95 (0.88–1.03)		1.51 (1.27–1.78)	
*Grade*			< .0001		< .0001		< .0001		0.4439
Grade 2	Grade 1	2.31 (2.15–2.48)		0.78 (0.45–1.37)		1.58 (1.50–1.66)		1.34 (0.77–2.33)	
≥ 3		4.58 (4.21–4.98)		1.72 (1.01–2.93)		1.80 (1.68–1.92)		1.40 (0.81–2.40)	
*Tumor location*			< .0001		0.0074		< .0001		< .0001
UIQ	UOQ	0.95 (0.88–1.04)		0.90 (0.75–1.09)		0.52 (0.48–0.56)		0.49 (0.39–0.61)	
LIQ		0.78 (0.69–0.89)		0.87 (0.67–1.13)		0.75 (0.68–0.83)		0.65 (0.48–0.87)	
LOQ		1.00 (0.90–1.11)		0.66 (0.49–0.88)		1.01 (0.94–1.10)		0.62 (0.46–0.83)	
Axillary tail		0.79 (0.48–1.27)		0.32 (0.08–1.34)		1.17 (0.86–1.59)		1.50 (0.67–3.35)	
Nipple/central portion		1.28 (1.11–1.47)		0.53 (0.31–0.91)		1.41 (1.27–1.57)		1.18 (0.79–1.75)	
Overlapping/NOS		0.94 (0.88–1.01)		0.81 (0.69–0.96)		0.88 (0.84–0.93)		0.76 (0.64–0.9)	
*Number of comorbidities*			0.0021		0.2062		0.0292		0.7746
1	0	1.04 (0.96–1.12)		1.03 (0.86–1.24)		1.06 (0.99–1.12)		1.05 (0.87–1.27)	
≥ 2		1.28 (1.11–1.46)		1.31 (0.97–1.76)		1.13 (1.01–1.27)		1.11 (0.79–1.55)	
*Race/ethnicity*			< .0001		0.0281		< .0001		0.1809
Hispanic	White	1.28 (1.13–1.45)		1.40 (1.06–1.85)		1.27 (1.15–1.40)		1.38 (1.02–1.86)	
Black		1.10 (1.00–1.20)		1.04 (0.88–1.23)		1.10 (1.02–1.18)		1.03 (0.87–1.23)	
Other		1.25 (1.09–1.45)		1.46 (1.02–2.11)		0.92 (0.81–1.05)		0.89 (0.57–1.40)	
Age group			< .0001		< .0001		< .0001		0.0783
≤ 50	> 50	1.18 (1.09–1.28)		1.42 (1.18–1.72)		1.26 (1.18–1.34)		1.19 (0.98–1.46)	
*Number of nodes examined*							< .0001		< .0001
2–3	1					1.38 (1.30–1.46)		1.43 (1.16–1.78)	
4–5						1.75 (1.63–1.88)		2.22 (1.74–2.83)	
≥ 6						6.17 (5.77–6.59)		9.6 (7.71–11.97)	

*HR* hormone receptor, *OR* odds ratio, *CI* confidence interval, *UIQ* upper inner quadrant, *UOQ* upper outer quadrant, *LIQ* lower inner quadrant, *LOQ* lower outer quadrant, *NOS* not otherwise specified

## Discussion

Multivariable analyses of clinical T1N0M0 ductal breast carcinoma patients who received BCS as their first definitive treatment revealed that the preoperative period (TTS) was a significant predictor of disease progression in HR-positive patients, with 8% and 30% higher odds of T-upstaging at ≥ 61 and ≥ 91 days TTS, respectively, and 17% higher odds of N-upstaging at ≥ 91 days TTS. Studies demonstrating a positive association between TTS and mortality risk among patients with early-stage breast cancer,^9^,^10^ as well as a positive association between TTS and upstaging of DCIS,^8^,^13^ raised the question of whether a prolonged preoperative interval is associated with measurable disease progression. However, earlier studies have not shown an association between TTS and upstaging in invasive disease.^11^–^13^ An institutional study enrolling clinically node-negative invasive breast cancer patients (*n* = 635) demonstrated no association between time from radiographic screening (mammography or sonography) to surgery (median 21 days, range 1–132 days) with tumor size or lymph node upstaging.^11^ Similarly, a comparison of serial sonographic images at the time of initial diagnosis and the day prior to surgery (median 31, range 8–78 days) among 323 unifocal invasive breast cancer patients concluded that time between diagnosis and surgery >30 days was not associated with tumor growth compared with time ≤ 30 days.^12^ This discrepancy may be attributable to several factors, including shorter median or range of TTS compared with our cohort (median 28 days in HR-positive and 27 days in HR-negative; range 8–180 days), differing classifications of TTS intervals, and inclusion of a broader range of disease (stage, histology, and HR status). A more recent study for the analysis of cT1-2N0 disease using the NCDB (*n* = 279,090) showed that TTS had no association with upstaging from clinical to pathologic prognostic stage after adjustment; however, a trend of increasing upstaging with prolonged TTS was seen among patients with cT1N0 disease in unadjusted analysis.^13^ One of the key differences in our study is the use of a selective cohort of anatomic cT1N0M0 ductal breast carcinoma patients who received BCS as their first definitive treatment. Since mastectomy is indicated for higher-risk disease (e.g. multifocal, multicentric, family history/genetics, etc.), patients who underwent mastectomy were excluded due to differing disease nature from those who receive BCS.^13^ In fact, mastectomy was a significant predictor of disease upstaging in other studies.^8^,^13^ Similarly, patients with non-ductal histology such as lobular carcinoma were excluded based on predicted ambiguity of radiographic tumor size measurement reported in lobular carcinoma in situ.^13^ Lastly, our cohort was categorized by TTS intervals of ≤ 30, 31–60, 61–90, and ≥ 91 days, with ≤ 30 days TTS as the reference category based on the observed statistically significant difference in overall mortality observed in each 30-day interval beyond ≤ 30 days TTS.^9^ Overall, our data highlight the importance of minimizing the preoperative period to avoid the potential risk of disease progression in HR-positive T1N0M0 ductal breast carcinoma.

Similar to upstaging trends reported in DCIS patients, our analysis recapitulated a positive association between upstaging and HER2 positivity, higher histology grade, and younger age in T1N0M0 ductal carcinomas.^17^–^20^ Our multivariable analysis showed that the nipple/central portion of the breast is significantly associated with increased risk of upstaging relative to tumors located in the upper outer quadrant for HR-positive tumors, presumably due to the convergence of mammary ducts at the nipple-areolar complex.^21^ The high density of fibroglandular tissue in this region results in heightened radiographic opacity that poses a challenge for sensitivity of mammographic detection in retroareolar masses.^21^ Another clinically significant finding of our study was age-related differences in upstaging. This may be attributable in part to the higher prevalence of aggressive subtypes or higher breast density among premenopausal women.^19^,^22^ Breast Imaging Reporting and Data System (BI-RADS) category 4 and 5 disease (as opposed to category 3) is reported to be consistently and significantly associated with upstaging of DCIS to invasive breast cancer.^6^ Greater breast density, defined by a higher percentage of radiographically dense epithelial and stromal tissue in the breast as opposed to radiographically lucent adipose tissue, both decreases mammographic sensitivity for detecting breast masses and is an independent risk factor for breast cancer regardless of the method by which it is detected.^23^,^24^ A cross-sectional meta-analysis of over 11,000 women found that mammographic density decreased with increasing age in both premenopausal and postmenopausal women, with the greatest reduction in mammographic density occurring at the time of menopausal transition.^25^

During the coronavirus disease 2019 (COVID-19) pandemic, 15% of cancer patients reported that their inpatient surgical procedures had been affected.^26^ Given the concern over the potentially negative survival impact of excessive surgical delays, the COVID-19 Breast Cancer Consortium published guidelines for operative prioritization. T1N0 estrogen receptor (ER)-positive/HER2-negative patients are categorized into priority level C1 and recommended neoadjuvant endocrine therapy (NET) in the context of delayed operation^27^ based on the comparable efficacy and overall survival rate of NET to adjuvant endocrine therapy in the GRETA trial.^28^ A NCDB cohort study showed that postmenopausal women with cT2-4c ER-positive breast cancer (*n* = 2,294) treated with NET were 1.6 times more likely to undergo BCS.^29^ Similarly, Minami et al. recently demonstrated that in patients with cT1-2N0 breast cancer, NET use did not impact stage or overall survival, supporting its safe use as a means of delaying surgery in patients with ER-positive breast cancer during the COVID-19 pandemic.^13^ Additionally, randomized studies have demonstrated an overall response rate to NET of >50% and an NET-induced conversion rate to BCS of >30%.^30^–^32^ The duration and type of NET have been suggested to affect the response rate.^33^ While the incremental benefit of NET is evident at a therapy duration of up to 8 months,^32^,^34^ studies of short treatment duration showed limited efficacy,^35^ as the response rate of breast cancer to NET is slow. The P024 trial, during which postmenopausal women with ER-positive stage II–III breast cancers (*n* = 228) underwent treatment with neoadjuvant letrozole or tamoxifen for 4 months prior to surgery, revealed a significantly higher overall response rate in patients treated with letrozole (55% vs. 36%).^30^,^36^ Despite efficacy in downsizing tumors and the favorable safety profile of NET,^31^,^36^ the rates of disease progression during NET range from about 5–25%,^30^–^32^ partly due to molecular mechanisms that contribute to endocrine resistance.^37^–^40^ Since the primary focus of randomized trials using NET has largely been to downstage clinical stage T2 or higher tumors,^30^,^34^,^35^ its effect on T1N0 breast cancer remains unknown. Since the current study focused on patients who underwent surgery as their primary cancer treatment and excluded NET-treated cases, our data are not generalizable to those who were treated with NET and experienced extended surgical delays during the COVID-19 pandemic. Further follow-up retrospective studies to evaluate the impact of NET on T1N0 HR-positive breast cancer patients using a dataset with capacity for data adjustment for NET responses will be critical in gaining insight into breast cancer management.

The current study has several strengths and limitations. The large cohort size, along with the ability to adjust for multiple tumor properties and demographic characteristics with exclusion of potentially confounding subpopulations, provides, for the first time, a valuable context for understanding of the association between surgical delays and measurable disease progression of both tumor size and nodal status in IDCs. To our surprise, after multivariable analysis, TTS-dependent upstaging remained significant in HR-positive patients, but not in HR-negative patients, even though the likelihood of T-upstaging among HR-negative patients was nearly double that among HR-positive patients (11.0% vs. 6.8%). Additionally, there was no interaction between TTS and HER2 status in any of the models, suggesting both luminal A (HR-positive/HER2-negative) and B (HR-positive/HER2-positive) subtypes are likely to be affected by a delay in surgery. In light of this insight, which seems opposed to current understanding of HR-positive disease as slow-proliferating and HR-negative disease as rapidly-proliferating,^41^,^42^ it is critical to address the mechanism underlying HR-specific TTS-associated upstaging. One main limitation includes the varying detection sensitivity^43^–^45^ of radiographic images utilized for clinical staging. Accurate tumor size measurement by mammogram and sonogram remains a challenge, especially in dense breast tissue,^46^,^47^ lobular carcinomas,^48^ and lesions under the nipple/areolar complex.^21^ Since the type of imaging modality and BI-RADS score are unavailable in the NCDB, further investigation with such variables to address the ambiguity of radiographic imaging is necessary to further determine the contribution of TTS to upstaging. Additionally, although we used a clinically selective subpopulation for our analysis, characterized by small early-stage tumors, to substantiate TTS-associated upstaging of invasive breast cancer, this does not negate the possibility of TTS-associated upstaging in higher-stage disease or disease with non-ductal histology.

## Conclusion

Overall, preoperative disease progression of clinical T1N0M0 ductal carcinoma patients was largely attributable to intrinsic tumor biology, including subtype, histology grade, and tumor location, and to demographic factors, including age, race/ethnicity, and comorbidities. However, TTS presented a modifiable factor that was significantly associated with both T- and N-upstaging among HR-positive clinical T1N0M0 ductal carcinoma patients, underscoring the importance of timely surgery.
